# Automatic quality assurance of radiotherapy treatment plans using Bayesian networks: A multi-institutional study

**DOI:** 10.3389/fonc.2023.1099994

**Published:** 2023-02-28

**Authors:** Petros Kalendralis, Samuel M. H. Luk, Richard Canters, Denis Eyssen, Ana Vaniqui, Cecile Wolfs, Lars Murrer, Wouter van Elmpt, Alan M. Kalet, Andre Dekker, Johan van Soest, Rianne Fijten, Catharina M. L. Zegers, Inigo Bermejo

**Affiliations:** ^1^ Department of Radiation Oncology (Maastro), GROW School for Oncology and Reproduction, Maastricht University Medical center+, Maastricht, Netherlands; ^2^ Department of Radiation Oncology, University of Vermont Medical Center, Burlington, VT, United States; ^3^ Department of Radiation Oncology, University of Washington Medical Center, Seattle, WA, United States; ^4^ Brightlands Institute for Smart digital Society (BISS), Faculty of Science and Engineering, Maastricht University, Heerlen, Netherlands

**Keywords:** radiotherapy, AI, Bayesian network, plan review, quality assurance

## Abstract

**Purpose:**

Artificial intelligence applications in radiation oncology have been the focus of study in the last decade. The introduction of automated and intelligent solutions for routine clinical tasks, such as treatment planning and quality assurance, has the potential to increase safety and efficiency of radiotherapy. In this work, we present a multi-institutional study across three different institutions internationally on a Bayesian network (BN)-based initial plan review assistive tool that alerts radiotherapy professionals for potential erroneous or suboptimal treatment plans.

**Methods:**

Clinical data were collected from the oncology information systems in three institutes in Europe (Maastro clinic - 8753 patients treated between 2012 and 2020) and the United States of America (University of Vermont Medical Center [UVMMC] - 2733 patients, University of Washington [UW] - 6180 patients, treated between 2018 and 2021). We trained the BN model to detect potential errors in radiotherapy treatment plans using different combinations of institutional data and performed single-site and cross-site validation with simulated plans with embedded errors. The simulated errors consisted of three different categories: i) patient setup, ii) treatment planning and iii) prescription. We also compared the strategy of using only diagnostic parameters or all variables as evidence for the BN. We evaluated the model performance utilizing the area under the receiver-operating characteristic curve (AUC).

**Results:**

The best network performance was observed when the BN model is trained and validated using the dataset in the same center. In particular, the testing and validation using UVMMC data has achieved an AUC of 0.92 with all parameters used as evidence. In cross-validation studies, we observed that the BN model performed better when it was trained and validated in institutes with similar technology and treatment protocols (for instance, when testing on UVMMC data, the model trained on UW data achieved an AUC of 0.84, compared with an AUC of 0.64 for the model trained on Maastro data). Also, combining training data from larger clinics (UW and Maastro clinic) and using it on smaller clinics (UVMMC) leads to satisfactory performance with an AUC of 0.85. Lastly, we found that in general the BN model performed better when all variables are considered as evidence.

**Conclusion:**

We have developed and validated a Bayesian network model to assist initial treatment plan review using multi-institutional data with different technology and clinical practices. The model has shown good performance even when trained on data from clinics with divergent profiles, suggesting that the model is able to adapt to different data distributions.

## 1 Introduction

Radiotherapy (RT) is a complex multidisciplinary procedure where different professionals are involved in the development and execution of a treatment plan (1). Each part of the RT workflow (2) is sensitive to errors that can have a negative impact and severe implications in the treatment outcome. For instance, radiation overdose to patients during the treatment delivery can lead to radiation toxicity, like inflammatory or autoimmune diseases (3). Current clinical RT care relies on a range of manual quality assurance (QA) tests to detect abnormalities and potential errors in radiotherapy processes (4, 5). With the rapidly advancing technology and complexity in RT, QA is a highly important task to provide high quality health care in radiation oncology.

In recent years, artificial intelligence (AI) techniques have been implemented in different parts of the RT workflow (6, 7). More specifically, AI has contributed to the automation and acceleration of the RT treatment planning procedure (8), organs at risk (OARs) delineation (9), and the development of decision support systems for patients’ treatment (10). For quality assurance, AI developments have been more limited (11). Efforts have been made on evaluating patient specific QA and machine QA using datasets based on (inter)national treatment protocols and safety guidelines (12, 13). AI can potentially improve the efficiency and efficacy of QA without increasing human resource needs (14).

This study focuses on improving the initial external beam radiation therapy plan review. In this QA procedure, different RT professionals are involved including medical physicists, radiation oncologists, and radiation technologists/dosimetrists. They check different technical, imaging, and dosimetric parameters of a treatment plan which are known from experience or literature to ensure the quality and safety of the treatment plan. International organizations such as the American Association of Physicists in Medicines (AAPM) created working task forces that are in charge of publishing QA guidelines (15). To automate the initial review process, most published studies implement such a guidelines-based approach (16, 17) Moreover, recent studies focused on the QA procedure are proposing promising AI-based applications (17–19). One of the reasons that the clinical introduction of AI-based treatment plan QA is lagging is the lack of reproducibility and external validation using data from multiple institutes (16).

To assist the initial plan review process, Luk et al. (17) proposed a Bayesian network (BN) model for the early detection of potential errors in the RT treatment plan. Based on different diagnostic, treatment planning, radiation dose prescription, and patient setup variables, Luk et al. (17) created an intelligent alert system that can warn medical physicists for potentially erroneous or suboptimal parameters in the RT treatment plan. The BN structure was created from a dependency-layered ontology (18, 20) and RT experts experience at the University of Washington [UW] (Seattle, WA, USA). BNs are probabilistic graphical models that model the interactions among a network of variables and can be used to estimate the probability of an event based on partial information. Their ability to deal with missing values (which is a common phenomenon in RT datasets) in combination with the intuitiveness of their probabilistic reasoning, make BNs an ideal method for decision support in radiation oncology (18). Compared to rules-based algorithms and checklists that have been developed to assist treatment plan review both in-house and commercially (21–31), the BN has the advantage of mimicking human reasoning and adapting to changes in clinical practice by updating the network model with new data (11).

The network created by Luk et al. has been externally validated by an independent European radiotherapy center (Maastro Clinic, Maastricht, The Netherlands) to assess its generalisability in a significantly different clinical setting, using a different patient cohort treated with different technology (treatment planning system and treatment delivery machine) (32).The results of the study (32) showed that whereas the network is reproducible and reusable by an independent institute, the performance of the model dropped when compared to that achieved on the development cohort, with variations in the different error categories that were simulated.

The goal of this study is to describe the development of an updated version of the error detection BN, by evaluating the performance of the BN including additional variables and connections. The updated version is based on the clinical expertise of RT professionals and clinical, treatment planning and dosimetric data from three different RT institutions in the United States and Europe: Maastro Clinic, UW, and The University of Vermont Medical Center [UVMMC].Following a standardized methodology for the data variables preprocessing and errors simulation, we aim to provide the RT community with a reproducible alert system for the early detection of errors that are observed in the routine clinical procedure of RT treatment planning.

## 2 Materials and methods

### 2.1 Model structure

**Figure 1:**
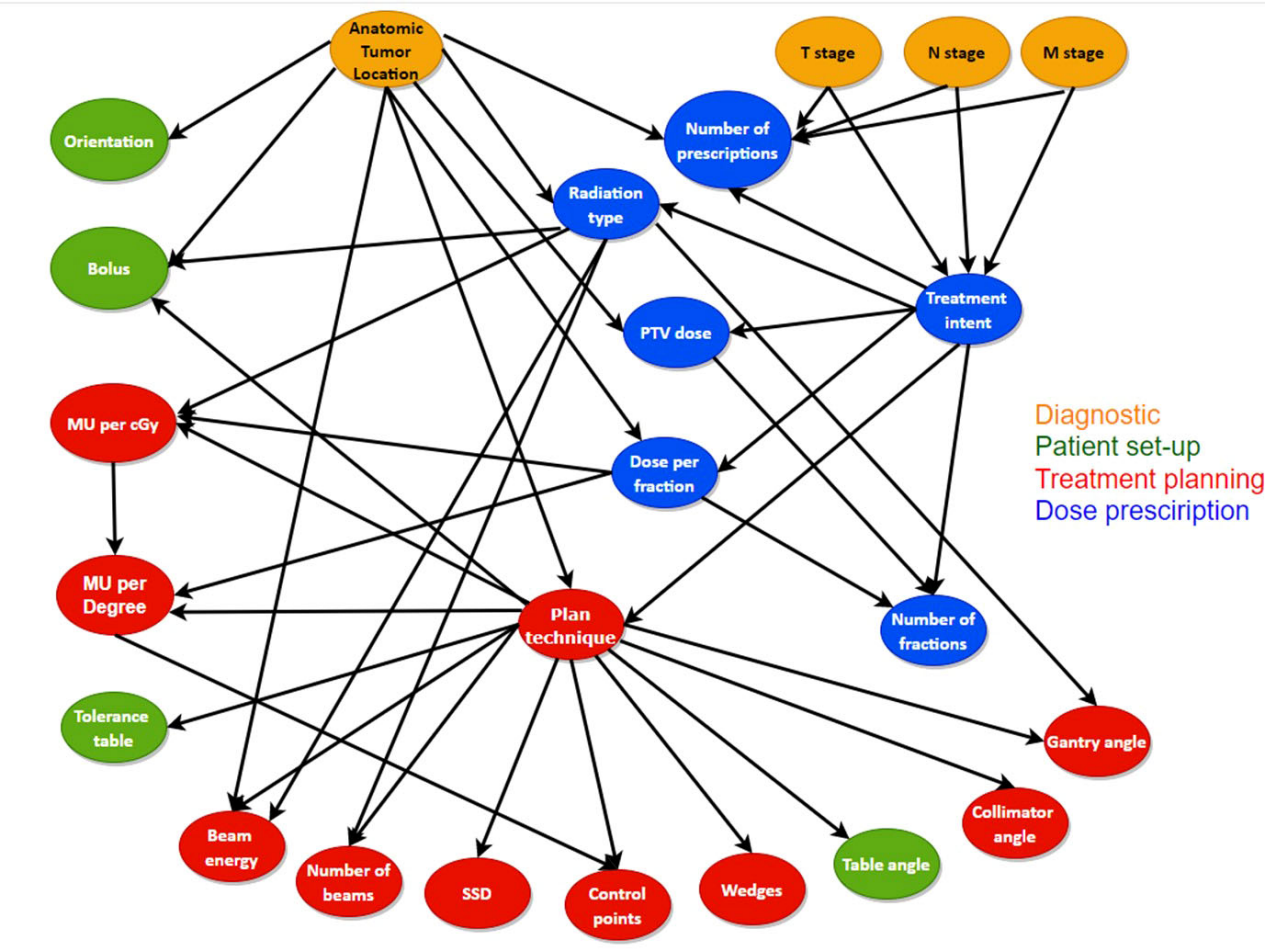
The structure and the connections between the different variables used for the new BN. The variables included in the different error categories as well as the diagnostic variables are represented with different colors.

With the BN structure created by Luk et al. (17) as the starting point, a new BN structure was created in collaboration with different experts such as medical physicists and radiation technologists/dosimetrists who are involved in the treatment planning procedure. We added two treatment plan variables to the network; 1) the involvement of the number of monitor units (MU) delivered for a certain gantry angle in a non-VMAT plan and 2) the delivered radiation dose per fraction in centi-gray (cGy). These two variables incorporate the plan complexity as part of the initial RT plan review in the network. We added links to the network structure if there was consensus amongst the experts interviewed with the goal of improving the model’s performance as well as making it more informative, reusable and interoperable with other radiotherapy centers. At the same time, we deleted setup equipment nodes from the original network due to the inconsistency between centers and difficulty in extracting information from mostly free text data. The new BN structure is shown in **Figure 1**, which includes 24 nodes and 41 edges with diagnostic parameters, prescription parameters, patient setup parameters and treatment planning parameters from a patient RT treatment plan.

### 2.2 Data acquisition

We used three different treatment plan datasets acquired from the relational database of the oncology information system (OIS) from three different institutions located in Europe (Maastro clinic-8753 patients treated between 2012 and 2020) and the United States of America (University of Vermont Medical Center - 2733 patients, University of Washington - 6180 patients, treated between 2018 and 2021). All data are anonymized and only the treatment plan variables that are included in the BN are extracted, as shown in the **Table S1** of the supplementary material. The different technical characteristics of the three institutions in terms of LINAC models and TPS are presented in **Table 1**.

**Table 1:** Technical characteristics of the three institutes.

Institutes/Technical characteristics	LINACs	Treatment planning system	Oncology Information system
MAASTRO	Varian	Eclipse	Aria
UW	Elekta	Raystation/Monaco	Mosaiq
UVMMC	Elekta	Pinnacle	Mosaiq

### 2.3 Datasets preparation

The terminology in the datasets was standardized between the three radiotherapy institutions. We also transformed the values into different “bins” to reduce the amount of potential states of each variable in BN. The variables and states that are used in the BN can be found in **Table S2** of the supplementary material. For training and testing purposes, we splitted the dataset of each institution to 80/20, where the 80% of data is used to train the BN, and the 20% of data is used for testing.

### 2.4 Error simulation

Due to the low number of registered errors in the incident reports of the clinics, we instead used simulated plans with embedded errors for testing and validation of the BN. Errors were simulated according to the high-risk failure modes discussed in the report of task group 275 of AAPM (15). The goal of the report was to provide recommendations on a physics plan and chart review using a risk-based approach. The failure modes that can be encountered by the BN were selected according to the needs of error alert probabilities of each institute.

We simulated errors in 5% in the testing dataset of the available treatment plans of each center based on the consultation of technical and clinical experts of each center and the real number of registered incidents/errors. The simulated errors can be classified into three different categories:

i) Patient setup,
ii) Treatment planning,
iii) Prescription.

As an indication of the simulated errors per category, we describe a few examples below. The first category consisted of simulated table angle errors altered by 10 degrees and errors such as the erroneous involvement of bolus during the delivery of RT. The second category of the simulated errors included planning errors such as LINAC gantry and collimator angles changed by 20-30 degrees and abnormally high/low plan complexity leading to unusual MU per cGy and MU per degree. Regarding the dose prescription category (third category), errors related to the fractionation scheme that was prescribed to the patients were simulated. Specifically, we selected to follow three approaches for the simulation of this error category, Initially, the combination between the dose per fraction and number of fractions was altered in order to have the same planning tumor volume (PTV) values for each treatment plan. The second approach of error simulation for this category consisted of simulations of different combinations between the PTV dose and the number of fractions with the dose per fraction stable. The third approach of the dose prescription errors included the simulation of different combinations between the PTV dose and the dose per fraction, while keeping the number of fractions stable. A classification of the errors simulated can be found in **Table 2** as well as with their detailed description in **Table S3** of the supplementary material. All the simulated error values were compliant with the data standardization framework we presented (ie. data binning), adjusted to the different treatment planning systems of each center.

**Table 2:** Error categories.

Patient set-up:Bolus, Patient orientation, Table angle, Tolerance table*	
Treatment planning: Beam energy, Radiation type, Number of beams, SSD, Collimator angle, Gantry angle, Treatment intent, Wedge*, MU/cGy,MU/degree	
Dose prescription: PTV dose, Number of fractions, Dose per fraction	

*Not applicable at Maastro.

### 2.5 Parametric learning

The Bayesian networks’ parameters were learnt with 80% of the data using the EM algorithm (33) implementation in Hugin 7.4, with a Laplace correction of the multiplicative inverse of the parent combinations of each node and a convergence threshold of 10^-4^.

### 2.6 Intended use

As in Luk et al (17) the intended use of the Bayesian network as a potential error detector and quality assurance on RT treatment plans is as follows: we instantiate some of the variables in the network as evidence and calculate the marginal probability of the value for each other variable in a given treatment plan. Potential errors or suboptimal treatment plan parameters are flagged if the marginal probability is under a certain threshold (referred to as anomaly threshold hereafter). For example, if the number of fractions in a particular plan is 25 for a patient with lung cancer, we calculate the probability of observing ‘Number of fractions’ = 25 after instantiating the ‘Anatomic tumor location’ node to ‘Lung’. If such a probability is lower than an anomaly threshold, e.g. < 0.05, we flag it as a potential error. The threshold is selected based on the practical experience at UW. For clinical use, the threshold should be determined by the local quality assurance team, with the tradeoff between increased false positive alert rates and missed potential errors in mind when threshold is increased and decreased respectively. In the original study, TNM staging, anatomic tumor location and the treatment intent variables were instantiated as they are diagnostic parameters which have been confirmed in other clinical procedures. In this study, we compare this strategy to the strategy where all other variables (that are not missing) are instantiated except the variable of interest, intending to compare the original study results.

### 2.7 Experimental setup

For the calculation of the marginal probabilities, the Java application programming interface (API) of Hugin Researcher 7.4 was used (34). The discriminative performance of the BN was assessed by calculating the area under the receiver-operating characteristic curve (ROC) curve (AUC) on the testing set. The ROC was calculated on all variables except TNM staging, anatomic tumor location and the treatment intent, which are assumed to be correct as mentioned, by calculating sensitivity and specificity for each possible value of the anomaly threshold.

Four different experiments were performed, which are described in **Table 3**, to evaluate the performance of the network in combinations of the three different centers by instantiating i) T, N, and M stages, anatomic tumor location and the treatment intent variables and ii) instantiating all the other variables.

**Table 3:** Experimental set-ups for the training and validation of the BN.

Experiment 1	Single site: Trained and tested on data in the same site
Experiment 2	Cross site: Trained on one site and tested on the other two
Experiment 3	Trained on two sites, tested on the third
Experiment 4	Trained and tested on data from the three sites

The code used for the data pre-processing steps, error simulation and the training/validation of the network can be found in the GitHub repository (MaastrichtU-CDS/projects_bn-rt-plan-qa: Bayesian network for error detection for radiotherapy planning (github.com)).

## 3 Results

### 3.1 Experiments

#### 3.1.1 BN single site approach: Training and testing on different dataset splits of the same center

**Figure 2:**
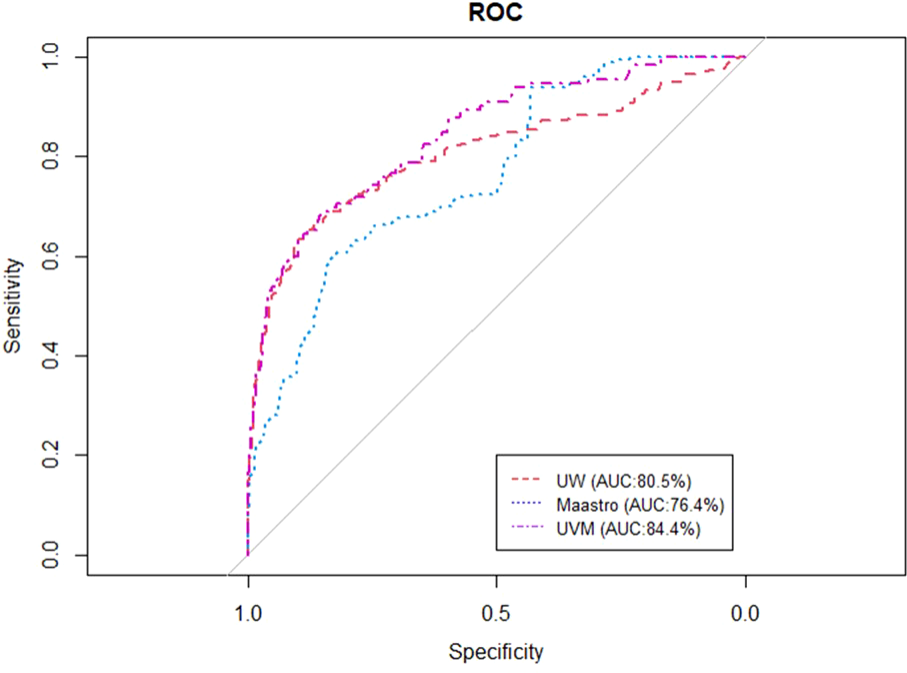
ROC curve of the BN using the single site validation approach (training and validation in one center) when instantiating anatomic tumor location, treatment intent and TNM stage.

**Figure 3:**
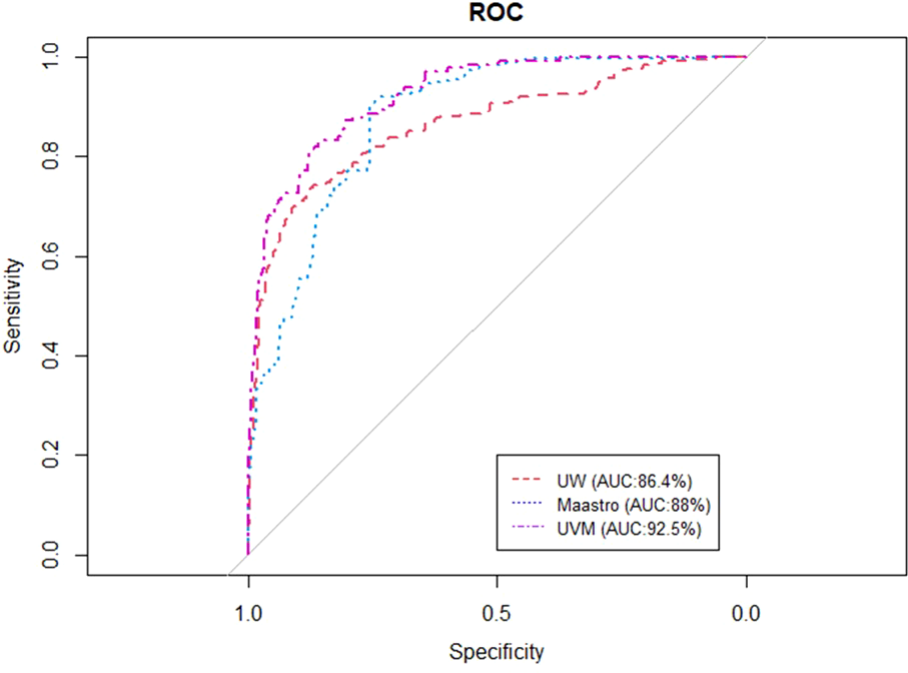
ROC curve of the BN using the single site validation approach (training and validation in one center) when instantiating all the variables of the BN.

**Figures 2**, **3** show the ROC and AUC values of the BN trained and tested with single institutional data. The highest performance was observed for the UVMMC center when instantiating all the variables of the network as well as when instantiating the variables of anatomic tumor location, TNM stage and treatment intent. However, the performance of the BN is in general better with the strategy of instantiating all variables.

To investigate the impact of each individual variable of the BN to the discrimination assessment, we calculated the AUC values on selected variables that included simulated errors. These AUC values describe the effectiveness of the BN on flagging errors in this particular variable of the BN among the three different centres. This overview can be found in **Table 4**.

**Table 4:** Individual AUC values of each BN variable in the case of training and testing in one center (Bolus data could not be extracted from UVMMC data).

Variable	Maastro		UW		UVMMC	
	Instantiating TNM stage, Anatomic tumor location and treatment intent (%)	Instantiating all *(%)*	*Instantiating TNM stage, Anatomic tumor location and treatment intent (%)*	Instantiating all *(%)*	Instantiating TNM stage, Anatomic tumor location and treatment intent (%)	Instantiating all *(%)*
Beam Energy	84.8	87.9	67	74.8	69.2	84.7
Bolus	78.5	79.1	57.9	59.9	–	–
Collimator Angle	80.5	80.5	96.7	96.9	94.6	95.2
Dose Per Fraction	60.4	73	85.9	85.9	84.7	90.6
Gantry Angle	79.8	79	85.3	86	86.1	84.2
MU Per cGy	60.2	86.9	75	83.4	80	96.4
MU Per Deg	99.5	100	93.7	99	88.9	99.4
Number of Beams	75.4	71.5	97.2	96.2	81.6	84.5
Number of Fractions	82.8	85.6	75.1	75.2	74	74
PTV Dose	78.5	83.2	90.3	90.2	94.2	94.2
Radiation Type	93.6	98.7	83.2	96.3	89.8	99.2
SSD	79.8	79	93.8	95.9	84.1	87.6
Table Angle	98.7	99.3	97.9	98	96.6	98.8
Overall	76.4	88	80.5	86.4	84.4	92.5

-, Not applicable.

#### 3.1.2 Cross site validation: BN trained on one center and tested on the other two

**Table 5** represents the AUC values of the BN using the cross site validation mode (trained with data from one institution and tested with other two institutional data). The highest performance on cross-site validation is using UW-trained network to test on the UVMMC testing dataset, while the worst performance is shown with testing UW data with Maastro-trained network.

**Table 5:** AUC values of the cross site testing of the BN.

Training/testing	Maastro		UVMMC		UW	
	Instantiating TNM stage, Anatomic tumor location and treatment intent (%)	Instantiating all *(%)*	Instantiating TNM stage, Anatomic tumor location and treatment intent (%)	Instantiating all *(%)*	Instantiating TNM stage, Anatomic tumor location and treatment intent (%)	Instantiating all *(%)*
Maastro.	76.4	88	62.7	63.8	57	58.5
UVMMC	68.3	64.2	84.4	92.5	72.3	75.3
UW	71.4	67.6	79.9	84.8	80.5	86.4

With gray color we have highlighted the results from the same site data.

**Table 6** shows the individual AUC values of each of the BN variables when instantiating all of them. The highest AUCs are using UW/UVMMC network to test Maastro table angle, using UW network to check UVMMC MU per degree, and UVMMC network on Maastro collimator angle, with AUC > 0.95 On the other hand, the Maastro network is not effectively checking MU per cGy, MU per degree and number of beams in UVMMC and UW dataset, showing AUCs below 0.5.

**Table 6:** Individual AUC values of each BN variable in the case of cross testing approach when instantiating all the BN variables (Bolus data could not be extracted from UVMMC data).

Trained -> Tested Variable	Maas -> UVMMC	Maas -> UVMMC	UW -> Maas	UW -> UWM	UVMMC ->Maas	UVMMC ->UW
Beam Energy	52.1	54	71.6	62	79.5	54.5
Bolus	–	52.7	72.1	–	72	54.2
Collimator Angle	82	64.3	88.4	84.8	95.2	88.8
Dose Per Fraction	58	73.9	61.3	62.3	74.6	74.3
Gantry Angle	56.9	61.6	67.9	81.3	84.5	72.5
MU Per cGy	57.5	41.7	72.6	89.7	69.6	76.4
MU Per Deg	48.9	42.9	85.8	95.7	58.6	91.1
Number of Beams	40.2	31.3	55.3	82.8	70.1	65.5
Number of Fractions	55.9	57.3	63.9	57	61.1	69.9
PTV Dose Rx	59.9	69	56.5	76.5	44.8	74
Radiation Type	79.5	66.9	74.1	97	68.3	78.7
SSD	69.3	67	78.1	70.8	59.6	90.2
Table Angle	67.1	77.4	98	91.2	99.6	96.1
Overall	63.8	58.5	67.6	84.8	64.2	75.3

-, Not applicable.

#### 3.1.3 Training in two institutions and validation in the remaining one

**Figure 4:**
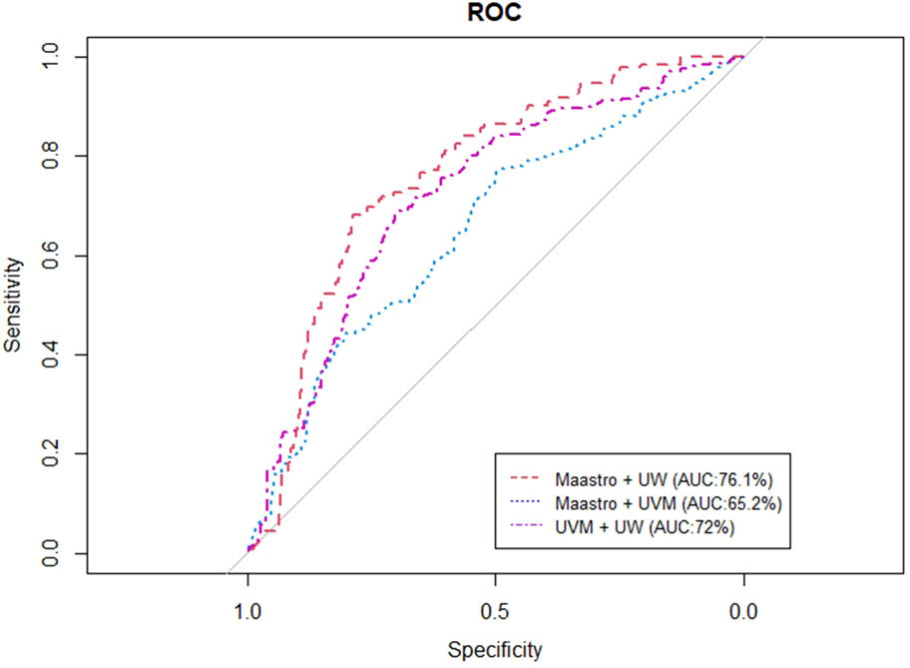
ROC curve of the BN performance when trained in two and validated in one center, when instantiating the three variables of anatomic tumor location, treatment intent and TNM stage.

**Figure 5:**
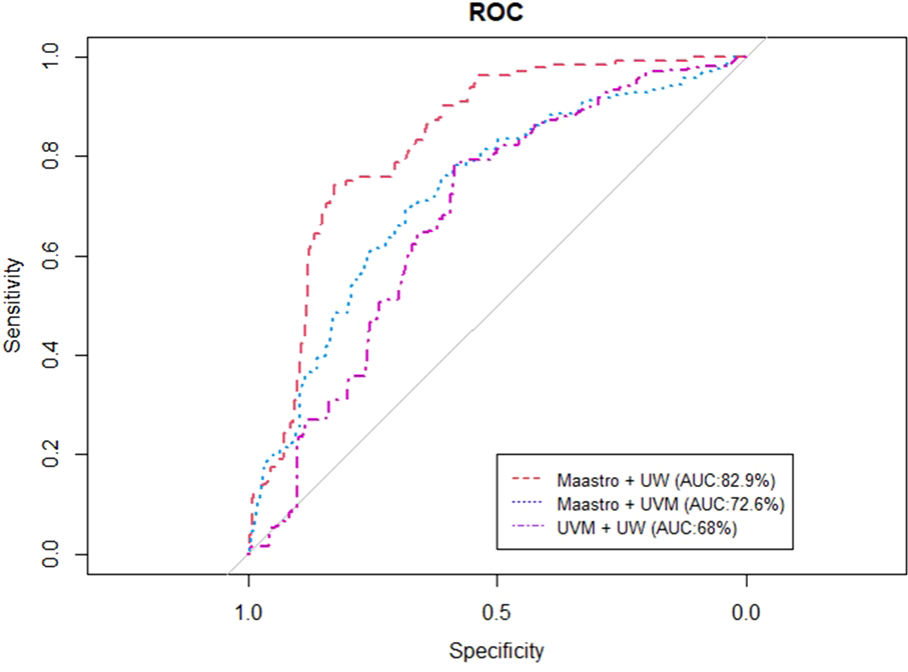
ROC curve of the BN performance when trained in two centers and validated in one, when instantiating all the BN variables.

**Figures 4**, **5** represent the performance of the BN when it is trained with data from two institutions and validated in the remaining one. The highest performance was observed for the training of the network at Maastro and UW centers and validated at UVMMC, in both of the cases of instantiating the three variables of anatomic tumor location, TNM stage and treatment intent (AUC=0.761) as well as when instantiating all of them (AUC=0.829).

#### 3.1.4 Training and validation in all three centers

**Figure 6:**
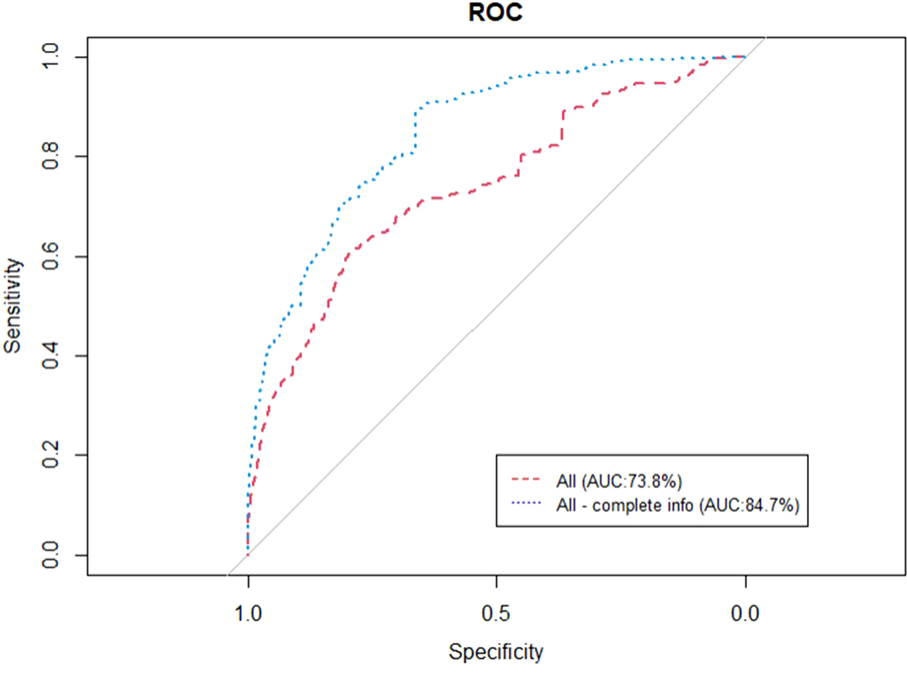
ROC curve of the BN performance when trained and validated in the three participating institutes.

**Figure 6** represents the performance of the BN when trained and validated in the three different participating centers, in both of the cases of instantiating the three variables of anatomic tumor location, TNM stage and treatment intent (AUC=0.738) as well as when instantiating all of them (AUC=0.847).

The individual AUC values of the BN when trained and validated in the three participating institutions are presented in **Table 7**. The highest performance was observed for the variable MU per degree in the case of instantiating all the variables (0.991).

**Table 7:** Individual AUC values of each BN variable in the case of training and testing the BN in all the three participating centers.

Variable	Instantiating TNM stage, Anatomic tumor location and treatment intent (%)	Instantiating all (%)
Beam Energy	71.7	82.2
Bolus	74.8	77.9
Collimator Angle	83.8	84.9
Dose Per Fraction	69.1	74.7
Gantry Angle	77.5	79.2
MU Per cGy	62.2	74
MU Per Deg	85.7	99.1
Number of Beams	78.2	76
Number of Fractions	76.9	78.2
PTV Dose Rx	77.9	79.3
Radiation Type	79.4	97.1
SSD	81.1	80.9
Table Angle	96.5	98.6
Overall	73.8	84.7

## 4 Discussion

In In this study, we established a multicentric approach to train and validate an AI-based system that can alert RT professionals of potential erroneous or suboptimal parameters in radiotherapy treatment plans as part of the initial plan review process. Our goal was to train and test the performance of the system in institutes with different treatment machines, treatment planning systems, oncology information systems and treatment protocols. The results showed that, while the performance of the BN alert system was good when trained and validated on the same sites, the performance is highly dependent on the similarity in terms of technology and clinical practice between training and testing datasets.

Specifically, the best performance was observed for UVMMC in the case of training and validating the network using training and testing dataset from the same center. Similar high performance was observed in the case of the BN validation using data from UVMMC in the cross site validation experimental setting. This is possibly due to the slightly narrower clinical profile at a regional single-site institution like UVMMC, which leads to a tighter distribution of the BN, versus a multi-site institution like UW or a large European institution like Maastro Clinic.

Another observation is that the BN performed better on American institutions (UW and UVMMC) when compared with Maastro clinic. It could be caused by the fact that most of the variables that are included in the current version of the BN were derived from the initial version of it in the study of Luk et al. (17) where the network was trained and validated in UW in North America. Another note on the single-institutional training and testing results is that instantiating all variables in the BN showed a better performance on flagging errors than the original proposal to instantiate only the diagnostic parameters (17). Individual variable AUCs showed consistent performance of the BN on different error categories, with the BN performing marginally worse in dose scheme in American institutions and number of beams in Maastro clinic.

The cross-site experimental set-up for the training and validation of the BN aimed to identify the optimal training strategy and the potential use of the BN trained from a data pool on a local clinic. We observed that the performance of the alert system was high in the case of training and validation when using datasets from the same center (single site approach). This can be explained by the fact that AI-based systems are highly dependent on the characteristics of the development datasets they are trained on (ie. training datasets) (35). Moreover, the heterogeneity of the treatment protocols adapted to the anatomical tumor sites as well as the different treatment techniques used in the three different institutions (even for the same tumor location).

In the cross-site validation, we found that the BN works better on mechanical treatment planning variables such as gantry angle and collimator angle, but less effective on dosimetric treatment planning variables such as number of beams, MU per degree and MU per cGy. The BN has also shown to be less effective on cross-checking plans in prescription parameters such as PTV dose on institutions in another continent (i.e., UVMMC and UW vs. Maastro). One possible explanation for the above-mentioned BN performance can be the differences in treatment machines (LINACs) and the treatment planning systems between the three centers, as well as the difference in technical characteristics and RT treatment protocols used between the two continents.

It is worth highlighting that for the third case of the experimental set-up (training of the network in two centers and validation in one), the performance of the BN was satisfactory in the case of combining training data from Maastro clinic and UW (and validated with UVMMC data). Also when the BN is trained with data from all three institutions, the performance is comparable to a single site approach (AUC 0.85 vs. 0.90), suggesting that a BN trained from a large data pool including divergent clinical profiles could be an effective tool for smaller clinics.

This study has several limitations; 1) the error simulation selection was based on the report for effective treatment planning review, clinical experience such as incident learning systems, and the errors of interest from clinical experts. It does not include all potential errors that could happen in an actual clinic, and the choice of the errors could affect the performance of the BN; 2) incomplete clinical profiles in the study. For example, there is a lack of data from North American institutions with Varian setups, which could be directly compared with our European counterpart, Maastro clinic, to improve the BN and further narrow down the potential causes of the results we observed.

This study also highlighted a few important steps to implement AI applications in clinics. First, it is important to create an AI model that could be applied to different clinical settings. Secondly, data standardization could greatly improve the generalizability and performance of the AI model. Ontologies (36) and community-created standardization schemes (e.g. AAPM TG-263) (37) are both potential tools to help achieve generalized AI models and standardized data. Our results also showed that the model can adapt to different data distributions, leading to generalizability to clinics with different profiles.

A pilot study on implementing the BN model was performed at UW (38, 39) (references). An in-house plan review assistive tool that combines a rules-based algorithm/checklist and the error detection BN was developed. The checklist reports a pass or fail of the rules to the physicists reviewing treatment plans and any failed rules would indicate action is needed on the plan. For the BN, the tool reports a pass, alert or warning parameter to the physicists when the particular parameter in the network has a conditional probability higher than the alert threshold, lower than the alert threshold but higher than the warning threshold, and lower than the warning threshold respectively. Usually an alert indicated that the parameter is uncommon from clinical data, but mostly correct, while the warning indicated that the plan parameter is rarely used in the clinic. The physicist used this information to determine if this plan parameter is suitable given the patient situation. The BN is updated annually according to the study result in Luk et al. Note that the tool is an assistive tool on initial plan review and does not replace any patient specific QA nor physicists reviewing the plan.

Future work will include the investigation of an alternative error simulation method that will be applicable and reproducible by other centers. Furthermore, the experimental set-up we used consists of four different levels of training and validation among the three different centers. As a next step, we aim to investigate another potential set-up including more international radiotherapy centers in order to test the reproducibility of the network from clinics with different technologies/dosimetrists and patient population characteristics. At the same time, we plan to expand the scope of BN to include additional treatment plan quality parameters such as DVH metrics, beam aperture size and irregularity. Finally, we will investigate the explicit modeling of divergent clinic profiles in the model, so that training data from similar clinics is prioritized when evaluating new treatment plans.

## 5 Conclusion

In conclusion, we presented an improved BN that has been validated in multiple institutions to alert RT professionals of potential erroneous or suboptimal parameters in radiotherapy treatment plans in the initial plan review process. The model has shown good performance even when trained on data from clinics with divergent profiles, but also that the performance is strongly dependent on the similarity between training and testing data in terms of technology and clinical practices.

## Data availability statement

The datasets presented in this article are not readily available because the authors of the study did not acquire the IRB approval in order to make them publicly available for the current stage of the study. Requests to access the datasets should be directed to petros.kalendralis@maastro.nl.

## Ethics statement

The studies involving human participants were reviewed and approved by IRB approval MAASTRO clinic W 19 10 00066. Written informed consent for participation was not required for this study in accordance with the national legislation and the institutional requirements.

## Author contributions

PK: Data pre-processing, errors simulation and contribution to the final structure of the Bayesian network for MAASTRO clinic. SL: Data pre-processing, errors simulation and contribution to the final structure of the Bayesian network for the University of Vermont Medical Center, Burlington, Vermont, United States. RC: Contribution to the final structure of the Bayesian network for MAASTRO clinic. DE: Contribution to the final structure of the Bayesian network for MAASTRO clinic. AV: Contribution to the final structure of the Bayesian network for MAASTRO clinic. CW: Contribution to the final structure of the Bayesian network for MAASTRO clinic. LM: Contribution to the final structure of the Bayesian network for MAASTRO clinic. WV: Contribution to the final structure of the Bayesian network for MAASTRO clinic. AK: Data pre-processing and contribution to the final structure of the Bayesian network for the department of Radiation Oncology, University of Washington Medical Center, Seattle, United States. AD: Assisted with manuscript proofreading. JV: Assisted with manuscript proofreading. RF: Assisted with manuscript proofreading. CZ: Data pre-processing, errors simulation, manuscript proofreading and contribution to the final structure of the Bayesian network for MAASTRO clinic. IB: Data pre-processing, errors simulation, statistical analysis and contribution to the final structure of the Bayesian network for MAASTRO clinic. All authors contributed to the article and approved the submitted version.
