# Supplementary material for: Automatic quality assurance of radiotherapy treatment plans using Bayesian networks: A multi-institutional study
Source: Front Oncol. 2023 Feb 28;13:1099994. doi: 10.3389/fonc.2023.1099994 (PMC10012863; doi:10.3389/fonc.2023.1099994)
Supplement: Supplementary file 1 [file Table_1.docx]

Automatic quality assurance of radiotherapy treatment plans using Bayesian Networks-A multi-institutional study

**Petros Kalendralis^*1^, Samuel M.H. Luk^*2^, Richard Canters^1^, Denis Eyssen^1^, Ana Vaniqui^1^, Cecile Wolfs^1^, Lars Murrer^1^, Wouter van Elmpt^1^, Alan M. Kalet^3^, Andre, Dekker^1,4^, Johan van Soest^4,1^, Rianne Fijten^1^, Catharina M. L. Zegers^1^, Inigo Bermejo^*1^**

1. Department of Radiation Oncology (Maastro), GROW School for Oncology and Reproduction, Maastricht University Medical center+, Maastricht, The Netherlands
2. Department of Radiation Oncology, University of Vermont Medical Center, Burlington, VT, United States
3. Department of Radiation Oncology, University of Washington Medical Center, Seattle, WA, United States
4. Brightlands Institute for Smart digital Society (BISS), Faculty of Science and Engineering, Maastricht University, Heerlen, The Netherlands

***Equal contribution**

**Correspondence:**Petros Kalendralis

Petros.Kalendralis@maastro.nl

# Supplementary Tables

| Table S1: Data items used from the three different clinics for the creation of Bayesian network | | |
| --- | --- | --- |
| Maastro clinic  TPS: EclipseOIS: ARIA, LINAC: Truebeam (Varian) | University of Vermont Medical CenterTPS: Pinnacle; OIS: Mosaiq; LINAC: Infinity (Elekta) | University of WashingtonTPS: RayStation; OIS: Mosaiq; LINAC: Infinity and Versa HD (Elekta) |
| Clinical T stage | | |
| Clinical N stage | | |
| Clinical M stage | | |
| Number of plan adaptations | | |
| Dose per fraction | | |
| Total dose | | |
| Number of fractions | | |
| Radiation type | | |
| Plan technique | | |
| Table angle | | |
| Number of beams | | |
| Wedge* | | |
| Control points | | |
| Source to surface distance (SSD) | | |
| Bolus | | |
| Gantry angle | | |
| Collimator angle | | |
| Beam energy | | |
| Table orientation | | |
| Tolerance table | | |
| Treatment intent | | |
| Monitor Units/cGy** | | |
| Anatomic tumor location | | |
| Monitor Units/deg** | | |
| *Do not exist in MAASTRO’s dataset,  **cGy=Centi Gray, **start and end arc angle for VMAT | | |

| Table S2: Variables states used for the creation of the BN by the three different participated centers | | | |
| --- | --- | --- | --- |
| Variable states/centers | Maastro | University of Vermont Medical Center | University of Washington |
| T stage | "T0" , "T1" , "T2", "T3", "T4", "Tis", "Tx" | | |
| N stage | "N0","N1" ,"N2", "N3", "Nx" | | |
| M stage | "M0", "M1", "Mx" | | |
| Number plan adaptations | 1, 2, 3, 4, >=5 | | |
| Dose per fraction | "[1001-1200]" ,"[1201-1400]", "[1401-1600]", "[151-200]", "[1601-1800]", "[1801-2000]","[2001-2500]","[201-250]", "[2501-3000]", "[251-300]", "[301-350]", "[351-400]", "[401-600]", "[601-800]", "[801-1000]", "<=150" | | |
| PTV dose | "[0-200]","[1001-1200]", "[1201-1400]", "[1401-1600]", "[1601-1800]", "[1801-2000]", "[2001-2200]", "[201-400]", "[2201-2400]", "[2401-2600]", "[2601-2800]", "[2801-3000]", "[3001-3200]", "[3201-3400]", "[3401-3600]", "[3601-3800]", "[3801-4000]", "[4001-4200]", "[401-600]", "[4201-4400]", "[4401-4600]", "[4601-4800]", "[4801-5000]", "[5001-5200]", "[5201-5400]", "[5401-5600]", "[5601-5800]", "[5801-6000]", "[6001-6200]", "[601-800]", "[6201-6400]", "[6401-6600]", "[6601-6800]", "[6801-7000]", "[7201-7400]", "[7601-7800]", "[801-1000]" | | |
| Number of fractions | 1-35 | 1-45 | 1-45 |
| Radiation type | "Electrons", "Photons" | “Electrons”, “Photons”, “HDR” | “Electrons”, “Photons”, “Protons”, Neutrons”, “HDR”, “LDR” |
| Plan technique | "IMRT", "VMAT" | “ConformalArc”, “ConformalPlan”, “VMAT”, “IMRT”, “SBRT”, “SRS”, HDRbrachytherapy” | “ConformalArc”, “ConformalPlan”, “VMAT”, “IMRT”, “SBRT”, “SRS”, HDRbrachytherapy”, “TBI”, “SIB” |
| Table angle | "[1-10]", "[11-20]", "[21-30]", "[281-290]", "[291-300]", "[31-40]", "[321-330]", "[331-340]", "[341-350]", "[351-359]", "[51-60]", "[61-70]", "[71-80]", "0", "90" | | |
| Number of beams | 1-15 | 1-20 | 1-20 |
| Wedge | No wedges are used at Maastro | ‘yes’, ‘no’ | ‘yes’, ‘no’ |
| Control points | "[101-120]", "[121-140]", "[141-160]", "[161-180]", "[2-20]", "[41-60]", "[61-80]", "[81-100]", "1" | | |
| Source to surface distance | "[101-105]", "[106-110]", "[111-115]", "[116-120]", "[80-85]", "[86-90]", "[91-95]", "[96-100]", "<80", ">120" | | |
| Bolus | "no", "yes" | | |
| Gantry angle | "[1-10]", "[101-110]", "[11-20]", "[111-120]", "[121-130]", "[131-140]", "[141-150]", "[151-160]", "[161-170]", "[171-179]", "[181-190]", "[191-200]", "[201-210]", "[21-30]", "[211-220]", "[221-230]", "[231-240]", "[241-250]", "[251-260]", "[261-269]", "[271-280]", "[281-290]", "[291-300]", "[301-310]", "[31-40]", "[311-320]", "[321-330]", "[331-340]", "[341-350]", "[351-359]", "[41-50]", "[51-60]", "[61-70]", "[71-80]", "[81-89]", "[91-100]", "0", "180", "270", "90" | | |
| Collimator angle | "[1-10]", "[101-110]", "[11-20]", "[111-120]", "[121-130]", "[131-140]", "[141-150]", "[151-160]", "[161-170]", "[171-179]", "[181-190]", "[191-200]", "[201-210]", "[21-30]", "[211-220]", "[221-230]", "[231-240]", "[241-250]", "[251-260]", "[261-269]", "[271-280]", "[281-290]", "[291-300]", "[301-310]", "[31-40]", "[311-320]", "[321-330]", "[331-340]", "[341-350]", "[351-359]", "[41-50]", "[51-60]", "[61-70]", "[71-80]", "[81-89]", "[91-100]", "0", "270", "360", "90" | | |
| Beam energy | 6 , 10 , 12 , 15 | 6, 9, 10, 12, 15, 18, Other | 6, 9, 10, 12, 15, 18, Other |
| Patient orientation | HeadIn-Supine | HeadIn-Supine, FeetIn-Supine, HeadIn-Prone, FeetIn-Prone, Unknown | HeadIn-Supine, FeetIn-Supine, HeadIn-Prone, FeetIn-Prone, HeadIn-RecumbentRight, HeadIn-RecumbentLeft, FeetIn-RecumbentRight, FeetIn-RecumbentLeft, Unknown |
| Tolerance table | Other | Photon-tight, Photon-loose, Electron, SRS/SBRT, Other | Photon-tight, Photon-loose, Electron, SRS/SBRT, Other |
| Treatment intent | "Curative", "NULL", "Palliative" | “Curative”, “Palliative”, “Neoadjuvant”, “Adjuvant”, “Induction” | “Curative”, “Palliative”, “Neoadjuvant”, “Adjuvant”, “Induction”, “BMTRegimen |
| MU/cGy | "[0-1]", "[1.1-1.5]", "[1.6-2]", "[2.1-2.5]", "[2.6-3]", "[3.1-3.5]", "[3.6-4]", "[4.1-4.5]", "[4.6-5]", ">5", "NULL" | | |
| MU/deg | "[0.1-1]", "[1.1-2]", "[2.1-3]", "[3.1-4]", "[4.1-5]", ">5", "0", "NULL" | | |

| Table S3: Categories of simulated errors with description | | |
| --- | --- | --- |
| Error category | Error | Examples |
| Patient set-up | Bolus | "Wrong bolus usage" |
| Treatment planning | Beam_Energy | "Beam energy should have been 6MV instead of 18" |
| Treatment planning | Radiation_Type | "Electrons should have been used instead of Photons" |
| Patient set-up | Patient orientation | "Patient should have been positioned to HeadIn-Prone" |
| Treatment planning | Number_of_Beams | "6 instead of 4" |
| Treatment planning | Source to surface distance | "100 instead of 80" |
| Treatment planning | Collimator angle | "20 instead of 10" |
| Treatment planning | Gantry angle | "90 instead of 80" |
| Patient set-up | Table angle | "10 instead of 0" |
| Treatment planning | Treatment intent | "Curative instead of palliative" |
| Treatment planning | Wedge* | "Wrong wedges usage" |
| Patient set-up | Tolerance table* | “Electron plan using Photon tolerance table” |
| Treatment planning | Monitor Units/cGy | "0 instead of 5" |
| Treatment planning | Monitor Units/degree | "5 instead of 0" |
| Dose prescription | PTV_Dose | 300 cGy x 20 fractions = 6000 cGy vs 200 cGy x 30 fractions = 6000 cGy |
| Dose prescription | Number_of_Fractions | 200 cGy x 20 fractions = 4000 cGy vs 300 cGy x 20 fractions = 6000 cGy |
| Dose prescription | Dose_Per_Fraction | 200 cGy x 30 fractions = 6000 cGy vs 200 cGy x 20 fractions = 4000 cGy |
| *Not applicable at Maastro | | |
